# The Association Between Significant Mitral Regurgitation and Atrial Fibrillation Recurrence Post-Ablation

**DOI:** 10.3390/jcm14207300

**Published:** 2025-10-16

**Authors:** Arni Gershman, Rivka Farkash, Amjad Abu-Salman, Mony Shuvy, Moshe Rav-Acha

**Affiliations:** 1 Braun School of Public Health and Community Medicine, Hebrew University-Hadassah, Jerusalem 9112001, Israel; 2 Medical Corps, Israel Defense Forces, Ramat Gan 5262000, Israel; 3 Cardiology Department, Shaare Zedek Medical Center, Jerusalem 9103102, Israel; 4 Medical Faculty, Hebrew University, Jerusalem 9103102, Israel

**Keywords:** mitral regurgitation, atrial fibrillation, ablation, recurrence, catheter ablation

## Abstract

**Background:** Atrial fibrillation (AF) is a common tachyarrhythmia associated with increased morbidity. AF frequently occurs alongside mitral regurgitation (MR). Although the impact of MR severity on AF is well proven, its effect on AF recurrence post-ablation is unclear and was the focus of our study. **Methods:** Retrospective single-center cohort of patients who underwent AF catheter ablation from 2014 to 2024. Pre-procedural transthoracic echocardiograms evaluated pre-ablation baseline MR severity. Patients with ‘significant’ MR (defined as moderate–severe or severe MR) were compared to those with ‘non-significant’ MR. Univariate Kaplan–Meier (KM) survival analysis, multivariable Cox proportional hazards models, and inverse probability treatment weighting (IPTW) method were applied to assess the association between baseline MR and AF recurrence post-ablation. **Results:** Among 444 patients undergoing AF ablation, 28 (6.3%) had ‘significant’ baseline MR. Over median follow-up of 19 months, 104 (23.4%) patients experienced AF recurrence. Univariate and KM survival analyses showed a non-significant trend for increased AF recurrence among patients with ‘significant’ MR. Applying KM analysis on balanced IPTW pseudo-population revealed robust association between ‘significant’ MR and AF recurrence post-ablation (HR = 2.41, 95% CI 1.80–3.22, *p* < 0.001). Multivariate analysis, performed on IPTW-adjusted pseudo-population, including age, gender, LA diameter, LVEF, and AF type, showed ‘significant’ MR to be independently associated with AF recurrence post-ablation (HR = 2.11, 95% CI 1.43–5.73, *p* = 0.003). **Conclusions:** Use of IPTW pseudo-population suggests a significant association between baseline MR severity, regardless of its etiology, and AF recurrence post-ablation. This association should be confirmed by future larger studies.

## 1. Introduction

Atrial fibrillation (AF) is the most common tachyarrhythmia [1]. AF is associated with increased morbidity, recurrent hospitalizations, increased stroke risk, and mortality [2,3]. Additionally, it carries a high burden on the health system, estimated to account for 1% of the National Health Service budget in the United Kingdom [2].

Many AF patients have concomitant mitral regurgitation (MR), which, by itself, is a highly prevalent valvular condition, with an estimated prevalence of 19% of the general population having ≥mild MR [4,5]. The main etiologies for MR are ‘primary’ ones, caused by valvular structural abnormalities, including degenerative, ischemic, and rheumatic etiologies, and ‘functional’ or secondary ones defined by preserved valve structure and caused mainly by annular dilatation resulting from left ventricular or atrial (LA) dilatation. MR is associated with an increased risk of death and heart failure and plays a prognostic role in many cardiac conditions [4,6].

The relationship between AF and MR is complex, and understanding this association is important for the management of patients with AF. It is well known that severe MR increases the risk of developing AF, due to structural and hemodynamic changes, including enlarged LA with increased filling pressures, which are known to be dominant risk factors for AF [7,8]. In addition to its impact on AF incidence, enlarged LA was shown to be associated with increased AF recurrence post-ablation [9]. Interestingly, in a recent study including 4466 participants from the 5th Copenhagen City Heart Study, even mild MR was associated with increased prevalence of AF [10]. This was further suggested in the ARIC trial, revealing that AF was more likely in heart failure (HF) patients with increased MR severity, regardless of HF subtype, but was more pronounced in HFpEF compared with HFrEF [11]. Notably, ARIC study analysis was based on a statistical methodology of IPTW, similar to that used in our study. Lastly, AF itself was shown to worsen functional MR severity by causing mitral annular dilatation, suggesting the interaction between AF and MR may be bidirectional [12,13].

As of today, a few studies have evaluated the impact of MR on AF ablation outcome, but these studies have mostly focused on functional MR [14,15], were based on a relatively small number of patients, and did not always assess the effect of MR severity but, rather, the presence or absence of MR [14]. To the best of our knowledge, only a single relatively small prior study evaluated the association between MR severity, including both primary and secondary etiologies, and AF recurrence post-ablation during a 1-year follow-up (F/U) duration [9]. In the current study, we aim to evaluate the role of baseline MR severity, regardless of its etiology, on AF recurrence following catheter AF ablation in a relatively large population with a longer follow-up duration. This will be done by comparison of AF recurrence post-ablation between patient subgroups with and without ‘significant’ baseline MR. Notably, if such an association is proved and found to have a cause-and-effect relation, one might think of MR reduction therapies (percutaneous or surgical) to increase AF ablation success.

## 2. Methods

This is a retrospective single-center cohort of patients with documented symptomatic AF who were referred for their first AF catheter ablation (index ablation) during the years 2014–2023 in The Jesselson Integrated Heart Center, Shaare Zedek Medical Center (SZMC), Jerusalem. Patients were included if they had undergone baseline trans-thoracic echocardiogram (TTE) within 3 months before the procedure and had a cardiology clinic follow-up visits (≥2) as well as post-ablation TTE within 3 months to 3 years post-ablation. Patients with prior ablations, prior cardiac surgery, or absence of cardiac follow-up visits after their index AF ablation were excluded. The study received approval from the IRB committee of SZMC. Notably, patients with baseline severe MR referred for AF ablation were mostly declined mitral valve surgery due to high surgical risk or patient preference.

### 2.1. Data Collection

Patient data were extracted from electronic medical records, including demographic information, clinical characteristics, comorbidities, echocardiographic parameters, laboratory results, and medications prescribed at discharge from index hospitalization. Notably, all cases had their pre-ablation clinical evaluation, ablation procedure, and post-ablation follow-up in SZMC. Accordingly, all patients had a routine post-ablation follow-up, including a 24-h ECG Holter within each clinic visit occurring at 1, 3, 6, and 12 months post-ablation along with 1-month external monitoring, at 6- and 12-month post-ablation. Moreover, all patients were instructed to document 12-lead ECGs whenever any arrhythmic clinical sign (such as palpitations) appeared. Implantable cardiac devices (if present) were interrogated at 6- and 12-month post-ablation visits.

### 2.2. Echocardiography

TTE examinations were performed as part of a routine clinical evaluation before ablation. Baseline MR severity was graded as mild, mild–moderate, moderate, moderate–severe, or severe based on the criteria of the European Society of Cardiology [16]. Other TTE parameters assessed included LA short axis diameter and left ventricular ejection fraction (LVEF). In our study, moderate–severe and severe MR were defined as ‘significant’ MR, while moderate or less MR were defined as ‘non-significant’ MR, in accordance with their definition in studies regarding candidates for percutaneous mitral valve repair [16,17]. Notably, not all TTEs were done in our center and MR etiology was not reported as a routine in TTE reports; therefore, we could not determine MR etiology in all cases.

### 2.3. Study Endpoint

The primary endpoint was AF recurrence, defined as any documented AF episode of >30 s, with or without symptoms, confirmed by a 12-lead electrocardiogram (ECG), 24 h ECG Holter, 1-month external ECG monitor, or pacemaker interrogation, occurring after the 3-month post-ablation ‘blanking’ period. The follow-up duration was calculated from the date of index ablation to the first documented AF recurrence, or the last follow-up clinical visit for patients without recurrence.

### 2.4. Statistical Methods

Continuous variables were presented as mean ± SD and/or median and interquartile range, depending on distribution, and categorical variables were presented by numbers and percentiles. The association of continuous variables with the study endpoint was evaluated via *t*- and Mann–Whitney tests (the choice between parametric or nonparametric test was dependent on the distribution of the continuous variable). Categorical variables’ association with the study endpoint was studied using chi squared or Fisher’s exact test. Kaplan–Meier (KM) survival analysis was used to assess the event-free (AF recurrence) survival over time according to MR severity at baseline.

In our historical cohort study, due to the relatively rare occurrence of the main exposure variable, namely ‘significant’ baseline MR, we faced difficulty in standard analyses, which may yield biased estimates due to poor covariate balance between the exposed and unexposed subgroups and low statistical power. Thus, we employed the inverse propensity score weighting (IPTW) method, which can help mitigate these issues by reweighting the sample to create a pseudo-population in which the distribution of baseline covariates is balanced and independent of the exposure status [18,19].

The IPTW method consists of two main steps. First, the probability of being exposed to the main exposure variable (‘significant’ baseline MR), namely the propensity score (PS), was calculated based on an individual’s demographic and clinical background characteristics and potential confounders, using a logistic regression model. In our study, the PS was estimated via a multivariable logistic regression, with MR severity as the independent variable. This model included background clinical characteristics that may be associated with ‘significant’ MR, including age, gender, history of CABG, history of PCI, ischemic heart disease, smoking status, and renal disease. The logistic model provided a PS for each case in the study. In the second step, weights were calculated for each case in the following way: individuals with ‘significant’ baseline MR (exposed) were weighted by the inverse of the PS, i.e., weight = 1/PS, while individuals without ‘significant’ baseline MR (unexposed) were weighted by the inverse of one minus the PS, i.e., 1/(1 − PS). Importantly, we used truncated weighting whereby extreme weights (below 1st and above 99th percentiles) were truncated, which is an alternative to conventional weighting used to handle outliers [19]. Applying these weights to the study population resulted in a pseudo-population with balanced distribution of covariates between the exposed and unexposed subgroups, namely the subgroups with and without ‘significant’ baseline MR [19,20]. The balance of covariates between these subgroups was assessed using standardized mean differences (SMD). A variable was considered balanced if the SMD was <0.1 [18,20].

On this pseudo-population, we repeated the univariate and KM analysis and applied multivariable Cox proportional hazards models to evaluate the independent relation between baseline MR severity and AF recurrence post-ablation, taking into account other possible confounders. The variables selected for adjustment in the multivariable analysis included age and gender as ‘universal parameters’, as well as parameters that were significantly associated with AF recurrence on the univariate analysis. We repeated the multivariable analysis to include LA diameter, LVEF, and AF type (persistent versus paroxysmal), which are known to be associated with AF recurrence post-ablation [21,22]. Hazard ratios (HR) with 95% confidence intervals (CI) were reported. An additional multivariable logistic model was applied on the weighted pseudo-population to assess the association of significant MR with 1-year AF recurrence. All tests were two-sided, and *p*-values < 0.05 were considered as significant. Analyses were carried out using SPSS software package version 30.0.0 (IBM, Armonk, NY, USA).

## 3. Results

**Figure 1:**
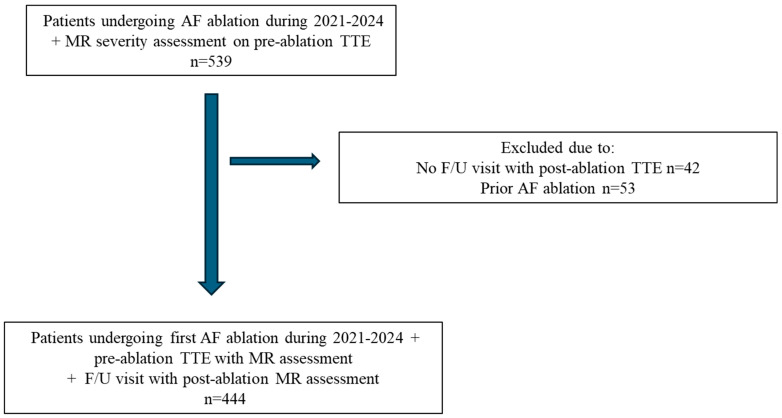
Inclusion/exclusion diagram.

There were 539 patients who underwent AF ablation at Shaare Zedek Medical Center during the years 2014–2023. All had a TTE done within 3 months prior to ablation, assessing baseline MR severity. After excluding patients with previous AF ablations, and those without an EP clinic follow-up visit, the study consisted of 444 patients (Figure 1). Of these, 59% had paroxysmal AF. The mean age was 64 ± 12 years; 60% were males, and 28 (6.3%) had ‘significant’ baseline MR. Index ablation was performed using cryoballoon in 318/444 (71.6%) cases and radio-frequency-based ablation in 126/444 (28.4%) cases. One hundred and four patients (23.4%) experienced AF recurrence within a median follow-up time of 19 (4–50) months post-ablation.

### 3.1. Baseline Characteristics and Their Association with AF Recurrence

The baseline characteristics of our study patients and their comparison with subgroups with and without AF recurrence are shown in Table 1. The patients in both subgroups were of similar age and had matching rate of comorbidities. The use of antiarrhythmic class Ic medication was more common among the subgroup without AF recurrence (24% vs. 12%, *p* = 0.011). Other medications at discharge were similar between the subgroups. During follow-up, 17 patients died. The mortality rate was similar between the subgroups (4.8 vs. 3.5%, *p* = 0.552).

Baseline TTE was performed in all patients 4 (1–42) days before ablation. The baseline TTE parameters are shown in Table 1. ‘Significant’ MR at baseline was present in 28 (6.3%) patients. The rate of ‘significant’ MR was higher among subgroup of patients with AF recurrence as compared to those without AF recurrence (8.6% and 5.6%, respectively, *p* = 0.13). Laboratory values were compared between the groups, revealing similar levels of hemoglobin (13.8 vs. 13.5 mg/dL, *p* = 0.327) and creatinine (1 vs. 0.9 mg/dL, *p* = 0.268). The median TSH levels were higher in patients who experienced AF recurrence (2.6 vs. 2.1 mIU/L, *p* = 0.049).

### 3.2. Association Between MR Severity and AF Recurrence, Based on IPTW Pseudo-Population

**Figure 2:**
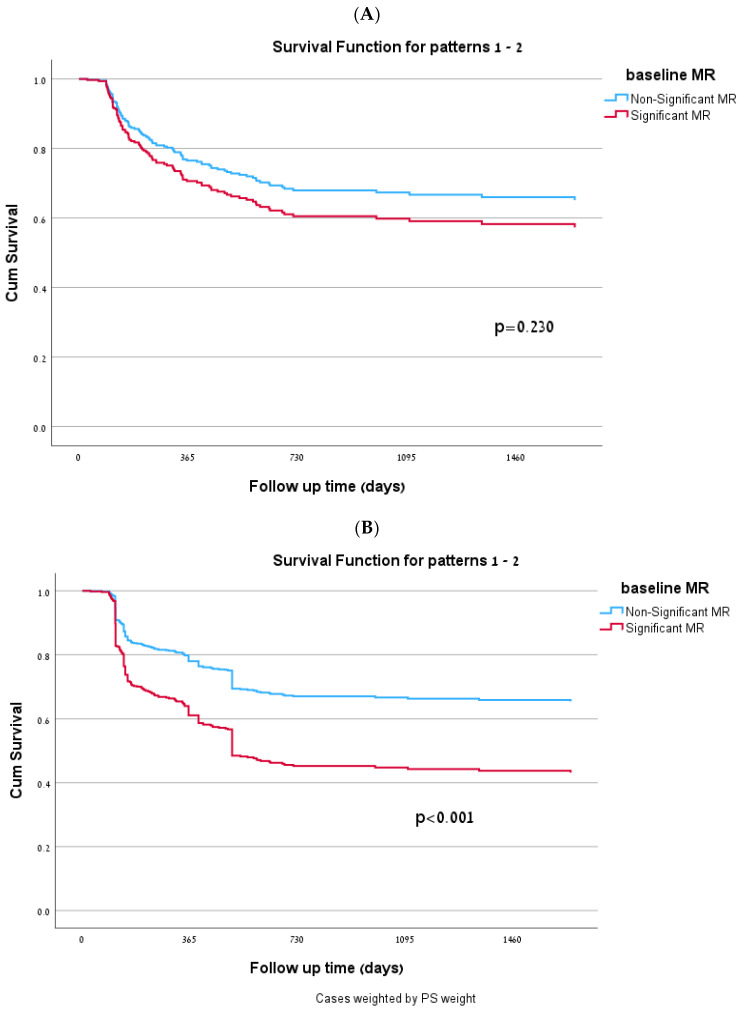
Kaplan–Meier curve of survival without AF recurrence in patients with and without ‘significant’ MR in the original dataset (**A**) and in the IPTW-adjusted pseudo-population, using truncated weighting (**B**).

On original raw-data KM analysis, a non-significant trend was observed between baseline MR severity and AF recurrence (p[log-rank] = 0.230; Figure 2A). Due to the small number of patients with ‘significant ‘MR (primary exposure subgroup), the IPTW approach was used, creating a pseudo-population with balanced covariates between the subgroups with and without ‘significant’ MR. Most of the covariates associated with MR severity were well balanced, as their post-weighting SMD was <0.1 (Supplementary Figure S1). Univariate analysis performed on the IPTW-weighted dataset showed ‘significant’ baseline MR to be associated with a higher rate of AF recurrence (HR = 2.16, 95% CI 1.68–2.77, *p* < 0.001). A KM analysis performed on the IPTW-adjusted dataset showed an increased incidence of AF recurrence among patients with ‘significant’ baseline MR as compared to those without ‘significant’ baseline MR (p[log-rank] < 0.001; Figure 2B).

To confirm the association of ‘significant’ baseline MR with AF recurrence, a multivariate Cox proportional hazards model was used and applied to the IPTW-weighted population. The model, including age and gender as universal parameters, as well as antiarrhythmic class Ic medication and baseline MR severity (both significantly associated with AF recurrence on univariate analysis), revealed that ‘significant’ baseline MR is independently and significantly associated with AF recurrence post-ablation (HR 2.41; 95% CI 1.80–3.22, *p* < 0.001; Table 2). We then repeated the multivariate model to include LA short axis (above or below 47 mm, which was the median LA short axis value in our study), LVEF (<50% versus ≥50%), and AF type (persistent versus PAF). In this model, ‘significant’ baseline MR was still significantly and independently associated with AF recurrence (HR 2.11; 95% CI 1.43–5.73. *p* = 0.003; Table 3).

## 4. Discussion

Our study aimed to evaluate the association between baseline MR severity and AF recurrence following AF ablation. The patient population consisted of 444 individuals who underwent their first AF ablation, with an evaluation of MR severity before ablation in all patients. An IPTW analysis, used to upweight the representation of the small subgroup with ‘significant’ MR and balance the influence of other covariates, suggests that ‘significant’ MR may be significantly associated with AF recurrence post-ablation. KM and multivariate analyses, both applied on the IPTW pseudo-population, suggest that ‘significant’ baseline MR is associated with AF recurrence post-ablation, independent of other parameters such as LA diameter, LVEF, and AF type.

To date, only a few prior studies have addressed this issue, and these differ in several ways from our work. Gertz et al. [9]. evaluated the impact of baseline MR severity on AF recurrence post-ablation among 190 AF patients with both primary and secondary MR, showing a higher rate of AF recurrence in patients with moderate or above MR within 1 year post-ablation. Yet, when performing multivariate analysis, only LA size was shown to be an independent predictor of recurrence, suggesting that MR severity is not independently associated with AF recurrence and its impact on AF recurrence might be mediated via LA size. In our larger size study with a longer follow-up duration of 19 months, MR severity was found to be associated with AF recurrence and independent of LA diameter, suggesting that, apart from MR effect on LA dilatation, MR per se may have an additional contribution to AF recurrence post-ablation. Recent studies have explored mechanisms by which MR may impact AF recurrence, independent of LA size. Possible mechanisms involve the geometric changes in the mitral valve apparatus itself, mitral annular calcification [23,24], and short posterior mitral leaflet [25].

Our findings align with prior studies [14,15], demonstrating a similar hazard ratio of MR for AF recurrence, although, in these studies, only patients with secondary/functional MR were included. Interestingly, other studies did not identify functional MR as a predictor of AF recurrence post-ablation [24]. Our study adds to the literature by evaluating the association of overall MR severity, regardless of its specific etiology, to AF ablation failure, in a large patient cohort with a relatively long follow-up duration.

Our study suggests that baseline MR may be considered another ‘clinical risk factor’ for AF ablation failure among other ‘formal’ risk factors (including LA size, AF duration, etc.). Thus, it may be prudent to assess MR severity when evaluating the overall risk for AF recurrence post-ablation.

According to the 2025 ESC/EACTS guidelines, the management of atrial MR should start with optimal treatment of AF, including appropriate rhythm- or rate-control strategies and anticoagulation [16]. Restoration of sinus rhythm may improve atrial size and mitral annular dynamics, as well as reducing MR severity, potentially delaying or obviating the need for valve intervention. If significant MR persists despite adequate AF management and the patient remains symptomatic, MV surgery is recommended, while transcatheter edge-to-edge repair (TEER) may be considered in selected patients (Class IIb). In those undergoing surgery, concomitant AF ablation should be considered as well [16]. Importantly, these recommendations, particularly regarding the role of TEER in AF patients with functional MR, are not based on randomized controlled trials, and prospective data are needed. The CAMERA-Pilot trial (NCT05846412), a randomized study directly comparing AF ablation per se versus TEER in patients with atrial MR, is expected to provide the first controlled evidence to guide therapeutic strategies in this population, helping to refine future guideline recommendations.

Importantly, our study aim was to evaluate baseline MR impact on recurrence of AF post-ablation in patients with MR of various degrees. For this aim, we retrospectively selected patients who did not undergo MV surgery (as explained in methods), but, rather, underwent catheter AF ablation. We do not suggest by any means that AF ablation is the treatment of choice for symptomatic AF patients with significant MR, as MV surgery with surgical ablation (ex. via MAZE procedure) is the treatment of choice per guidelines [16]. However, we do recommend future prospective randomized studies, specifically for patients with AF and atrial functional MR, to compare between the effect of AF ablation versus concomitant AF ablation and MV repair. Nevertheless, we should emphasize that, at the current stage, MV surgery is the preferred treatment for patients with severe MR, with or without AF.

The baseline characteristics of the study population, including age, gender, and various comorbidities, such as ischemic heart disease, hypertension, diabetes mellitus, and heart failure, were similar among those with and without AF recurrence post-ablation. The higher use of anti-arrhythmic class Ic medications in patients without AF recurrence may suggest these patients were healthier overall, as Ic anti-arrhythmic medications are usually given to healthier patients with preserved LV function and no ischemic background. Accordingly, this may explain their apparently strong protective effect from recurrent AF (HR of 0.17 in Table 2) given that, in clinical practice, they serve as a marker for structurally normal hearts. Notably, patients with AF recurrence had higher median TSH levels. Similar results were found by other studies as well [26,27,28], revealing that a part of the well-known association of hyperthyroidism with AF, hypothyroidism may also be associated with AF in general and specifically with AF recurrence post-ablation [27]. Mechanistically, animal studies suggest that hypothyroidism may be related to atrial fibrosis [28], which is considered a substrate for AF perpetuation and recurrence post-ablation [29,30,31].

Both MR (at least functional) and AF are strongly associated with the uprising concept of atrial cardiomyopathy (ACM), involving complex structural, functional, and electrical remodeling of atrial myocardium [29,30]. The clinical characteristics of these changes include the following: increased epicardial adipose tissue and atrial myocardial fibrosis, which could both be visualized and quantified well by modern MRI examinations; atrial mechanical loss of function, leading to mitral annular dilatation, resulting in functional atrial MR and TR; a tendency for increased thrombogenesis; and atrial arrhythmia, mainly AF [29,30]. Indeed, AF is no longer considered a ‘pure’ electrical arrhythmia but, rather, a prototype arrhythmia of ACM, resulting from numerous structural and electrical abnormalities within the atria [31]. Notably, AF could result from ACM but could also accelerate ACM via multiple mechanisms including atrial fibrosis, atrial and annular dilatation with increasing functional MR, and multiple electrical channel remodeling [29,31]. Accordingly, AF ablation may reverse some of these electrical and structural changes induced by AF and, thus, may ameliorate the degree of ACM [30]. On the other hand, the existence of other causes for ACM, such as MR, may still promote ACM despite ablation, eventually leading to AF recurrence. Thus, the association between MR severity and AF recurrence post-ablation could be easily conceptualized.

The IPTW approach used in this study has gained increasing popularity in observational studies [20], including in the field of cardiology [11,19]. It is mainly used to balance baseline patient characteristics or covariates, thus estimating the net treatment effect between treated and control subjects in a weighted sample. It is particularly relevant in observational studies, where confounding factors can present a significant challenge due to the lack of randomization. Using this method, a pseudo-population was created, where covariates are balanced between patients with and without ‘significant’ MR. This upweighted pseudo-population enabled us to estimate the impact of ‘significant’ MR on AF recurrence post-ablation.

### Limitations

This study has several major limitations. (1) The observational nature of the study limits our ability to establish causality, and, thus, our study could only suggest an association between MR severity and AF recurrence post-ablation. Moreover, we cannot rule out a potential selection bias, resulting from the study’s retrospective nature. For example, the fact that patients with significant MR underwent AF ablation may represent a selection bias for sicker patients who could not undergo MV surgery. (2) The small number of patients with ‘significant’ MR (main exposure parameter) in the original dataset. We used the IPTW method to upweight the representation of our exposure subgroup and to reduce selection bias by effectively balancing other covariates. However, IPTW analysis may be impacted by large weight variations when handling a small exposure subgroup. We mitigated this issue by truncating extreme weights and by verifying balanced covariates among subgroups with and without ‘significant’ MR. The balancing of covariates was done via standardized mean difference (SMD) analysis of the main covariates associated with the study exposure variable, revealing very small SMD post-weighting. (3) MR severity was determined by a TTE which was done as a routine prior to ablation and not conducted as part of a preplanned study. Thus, MR severity estimation did not always involve the calculation of all guideline-recommended TTE parameters for MR severity assessment, and we appreciate that some borderline cases might have been misclassified. Nevertheless, given that, in our work, we did not evaluate MR severity as a continuous parameter but, rather, split the patients between moderate–severe or severe MR versus moderate or less MR, this routine TTE should usually suffice. (4) The pre-ablation TTEs were done within 3 months before the ablation, enabling significant changes in volume overload and other conditions influencing MR severity. Thus, we cannot rule out MR severity changes that may have occurred just prior to the ablation. Notably, however, the actual median time of the TTE before ablation was 4 days, minimizing the chance for significant hemodynamic changes. Nevertheless, these changes could still have occurred, especially given the clinical practice of treatment optimization prior to procedure (treating patients with diuretics and blood pressure medications just prior to ablation), resulting in MR attenuation. (5) Not all TTEs were done in our center and MR etiology is not reported as a routine in TTE reports; therefore, we could not revise all TTEs and determine MR etiology in all cases. Nevertheless, as we wrote throughout the text, our aim was to assess the association of baseline MR severity, regardless of its etiology, to AF recurrence post-ablation. (6) Since LA volumes were not routinely evaluated, we considered LA diameter to represent LA size, acknowledging this assumption is not accurate. (7) We could not adjust our results for AF-ablation-related factors, which are known to contribute to AF recurrence post-ablation, because not all patients had the same ablation technique and not all patients with AF recurrence underwent redo ablation with mapping of PV gaps and other potential AF substrates.

## 5. Conclusions

Our study suggests that ‘significant’ baseline MR is associated with AF recurrence post-ablation, and that this association is independent of other clinical parameters known to impact AF recurrence, including LA diameter, LVEF, and AF type. Notably, our results were dependent on the IPTW balancing approach, with the limitations accompanying the use of such an approach, especially in a small exposure dataset. Accordingly, our study results should be confirmed by larger-scale randomized studies. Further studies are needed to evaluate for a causal relation between MR severity and AF ablation success. Confirmation of such a causal relation may have important clinical relevance, as MR severity would then be added to the list of ‘formal’ risk factors known to impact AF ablation failure, with the opportunity to assess the efficacy of mitral valve repair techniques in promoting AF ablation success.

## Figures and Tables

**Table 1:** Baseline characteristics based on raw data.

Variable	Overall Population (n = 444)	AF Recurrence (n = 104)	No AF Recurrence (n = 340)	*p* Value
**Clinical parameters**				
Age (y)	64 ± 12	63 ± 12	64 ± 12	0.743
Gender (male)	267 (60%)	66 (63.5%)	201 (59.1%)	0.429
Paroxysmal AF *	251 (59.2%)	53 (53.5%)	198 (60.9%)	0.191
**Ablation technique & post-ablation F/U** **				
Cryoballoon ablation	318 (71.6%)	76 (73.1%)	242 (71.2%)	0.706
24-h ECG Holter **	3.8 [2.9–4.2]	3.7 [2.6–4.1]	3.9 [2.7–4.4]	0.963
**Comorbidities**				
IHD	63 (15%)	16 (15.4%)	47 (16.8%)	0.682
Hypertension	262 (61%)	61 (58.7%)	201 (59.1%)	0.953
Diabetes mellitus	88 (20%)	16 (15.4%)	72 (21.2%)	0.197
Smoker	54 (13%)	8 (7.7%)	46 (13.5%)	0.112
Hyperlipidemia	133 (31%)	24 (23.1%)	109 (32.1%)	0.069
CHF	49 (11%)	11 (10.6%)	38 (11.2%)	0.870
TIA/Stroke	35 (8%)	8 (7.7%)	27 (7.9%)	0.939
CHA_2_DS_2_-VASc Score ≥ 3	163 (38%)	38 (36.5%)	125 (36.8%)	0.979
**Medications at discharge**				
Beta blockers	222 (51%)	52 (52%)	170 (50%)	0.982
Diuretics	74 (17%)	20 (19.2%)	54 (15.9%)	0.415
ACEi	81 (19%)	18 (17.3%)	63 (18.5%)	0.785
ARBs	40 (9%)	8 (7.7%)	32 (9.4%)	0.596
MRA	50 (13%)	11 (10.6%)	39 (11.5%)	0.955
Antiarrhythmic class III	25 (6%)	7 (6.7%)	18 (5.3%)	0.480
Antiarrhythmic class Ic	83 (21%)	10 (9.6%)	73 (21.5%)	0.011
Follow-up (days)	574 (127–1515)	869 (537–1503)	452 (76–1458)	<0.001
**TTE parameters**				
MR severity				0.843
None	86 (18.3%)	17 (16.3%)	69 (20.3%)	
Mild	187 (42.1%)	45 (43.3%)	142 (41.7%)	
Mild–moderate	89 (20%)	19 (18.3%)	70 (20.6%)	
Moderate	54 (12.2%)	14 (13.5%)	40 (11.8%)	
Moderate–severe	22 (5%)	7 (6.7%)	15 (4.4%)	
Severe	6 (1.3%)	2 (1.9%)	4 (1.2%)	
‘Significant’ MR (moderate– severe and severe)	28 (6.3%)	9 (8.6%)	19 (5.6%)	0.130
TTE timing before ablation (days)	4 (1–42)	3 (1–29)	4 (1–42)	0.601
LA short axis (cm)	4.5 [4.1–4.9]	4.7 [4.0–5.0]	4.5 [4.1–4.9]	0.367
EF				0.574
<40%	37 (10%)	9 (8.6%)	28 (8.2%)	
40–50%	50 (13%)	14 (13.4%)	36 (10.6%)	
50%≤	298 (77%)	64 (61.5%)	234 (68.8%)	

Data are shown as mean ± SD, no. (%) or median [IQR]; IHD, ischemic heart disease; CHF, congestive heart failure; TIA, transient ischemic attack; ACEi, angiotensin-converting enzyme inhibitors; ARBs, angiotensin receptor blockers; MRA, mineralocorticoid receptor antagonist; TTE, transthoracic echocardiogram; LA, left atrium; EF, ejection fraction. * Data regarding AF type (persistent versus paroxysmal) were available for 424/444 (95.5%) of the patients. ** Number of 24 h ECG Holters evaluated within 1-year post-ablation F/U (follow-up).

**Table 2:** Cox proportional hazards model for AF recurrence based on IPTW-adjusted dataset, using truncated weighting.

Variable	HR	95% Confidence Interval	*p* Value
Significant MR	2.41	1.80–3.22	<0.001
Antiarrhythmic class Ic	0.17	0.10–0.33	<0.001
Age (y)	0.97	0.95–0.98	<0.001
Gender (female)	0.68	0.48–0.95	0.026

**Table 3:** Cox proportional hazards model for AF recurrence including LA diameter, LVEF, and AF type based on IPTW-adjusted dataset, using truncated weighting.

Variable	HR	95% Confidence Interval	*p* Value
Significant MR	2.11	1.43–5.73	0.003
LA short axis (≥47 mm) *	1.71	1.03–2.80	0.058
Persistent AF versus PAF	1.36	0.78–3.57	0.142
EF < 50%	2.26	0.93–5.51	0.075
Antiarrhythmic class Ic	0.39	0.11–0.95	0.144
Age (y)	0.96	0.94–0.98	0.057
Gender	0.53	0.18–1.03	0.153

* LA short axis ≥median versus <median value in our study, which was 47 mm.

## Data Availability

The data presented in this study are available upon request from the corresponding authors due to privacy and legal restrictions made by Shaare Zedek Medical Center.

## Supplementary Materials

The following supporting information can be downloaded at: https://www.mdpi.com/article/10.3390/jcm14207300/s1, Figure S1: Standard mean difference of potential cofounders before and after IPTW adjustment. A variable with SMD ~0.1 after IPTW adjustment is considered to be well balanced; Table S1: Logistic regression model for 1-year AF recurrence based on IPTW-adjusted dataset.
