# Supplementary material for: The Association Between Significant Mitral Regurgitation and Atrial Fibrillation Recurrence Post-Ablation
Source: J Clin Med. 2025 Oct 16;14(20):7300. doi: 10.3390/jcm14207300 (PMC12565141; doi:10.3390/jcm14207300)

Supp Table S1: Logistic regression model for 1-year AF recurrence based on IPTW-adjusted dataset

**Variable**	**OR**	**95% Confidence Interval**	**P value**
Significant MR	2.36	1. 58- 3.53	< .001
Antiarrhythmic class Ic	0.11	0.05 – 0.25	< .001
Age (y)	0.98	0.96 - 1	0.061
Gender (female)	0.51	0.33 - 0.79	0.002

Supp Figure S1: Standard mean difference of potential cofounders before and after IPTW adjustment. A variable with SMD ~ 0.1 after IPTW adjustment is considered to be well balanced.

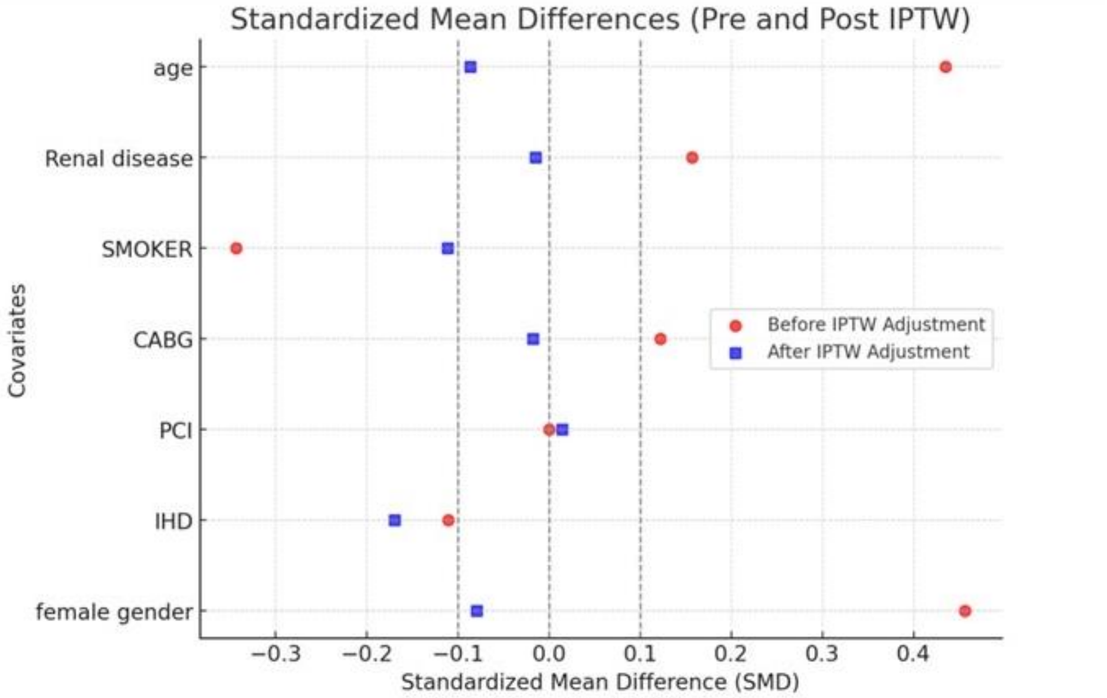

Supplement: Supplementary file 1 [file jcm-14-07300-s001.zip › jcm-3859960-supplementary.pdf]
